# Revisiting stigmergy in light of multi-functional, biogenic, termite structures as communication channel

**DOI:** 10.1016/j.csbj.2020.08.012

**Published:** 2020-08-19

**Authors:** Sebastian Oberst, Joseph C.S. Lai, Richard Martin, Benjamin J. Halkon, Mohammad Saadatfar, Theodore A. Evans

**Affiliations:** a Centre for Audio, Acoustics and Vibration, Faculty of Engineering and IT, University of Technology Sydney, 15 Broadway, Ultimo, NSW 2007, Australia; b School of Engineering and IT, University of New South Wales Canberra, Northcott Dr, Campbell ACT 2612, Australia; c Department of Applied Mathematics, Australian National University, 58-60 Mills Road, Canberra, ACT 2601, Australia; d School of Biological Sciences, The University of Western Australia, 35 Stirling Hwy, Crawley, WA 6009, Australia

**Keywords:** Termite structures, Complexity, Superorganism, Vibrational communication, Biotremology, Functional materials

## Abstract

Termite mounds are fascinating because of their intriguing composition of numerous geometric shapes and materials. However, little is known about these structures, or of their functionalities. Most research has been on the basic composition of mounds compared with surrounding soils. There has been some targeted research on the thermoregulation and ventilation of the mounds of a few species of fungi-growing termites, which has generated considerable interest from human architecture. Otherwise, research on termite mounds has been scattered, with little work on their explicit properties.

This review is focused on how termites design and build functional structures as nest, nursery and food storage; for thermoregulation and climatisation; as defence, shelter and refuge; as a foraging tool or building material; and for colony communication, either as in indirect communication (stigmergy) or as an information channel essential for direct communication through vibrations (biotremology).

Our analysis shows that systematic research is required to study the properties of these structures such as porosity and material composition. High resolution computer tomography in combination with nonlinear dynamics and methods from computational intelligence may provide breakthroughs in unveiling the secrets of termite behaviour and their mounds. In particular, the examination of dynamic and wave propagation properties of termite-built structures in combination with a detailed signal analysis of termite activities is required to better understand the interplay between termites and their nest as superorganism. How termite structures serve as defence in the form of disguising acoustic and vibration signals from detection by predators, and what role local and global vibration synchronisation plays for building are open questions that need to be addressed to provide insights into how termites utilise materials to thrive in a world of predators and competitors.

## 1 Introduction to termitology

**Fig. 1:**
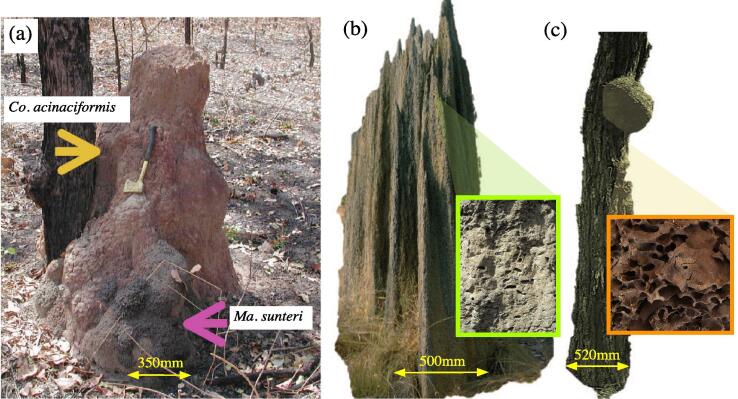
Termite nests of (a) *Coptotermes acinaciformis* with commensurate termite species *Macrognathotermes sunteri* attached to its host mound; Berrimah, Northern Territories (photo credits: Sebastian Oberst, 2011); (b) *Amitermes meridionalis*, the ”magnetic” or ”compass” termite, mound-building, hypogeal species [3] (Arnheim, Northern Territories, Australia CSIRO picture collection; photo credits: Coppi, 1992), and (c) a tree-nest of *Nasutitermes walkeri*, arboreal, higher termites (dead-wood feeding [18]), Warrambungle National Park, New South Wales, Australia (photo credits: Sebastian Oberst, 2018). Inserts show (b) a hard, outer shell and (c) a filigree inner structure.

Termites are eusocial cockroaches [1], many of which eat wood and show cryptic behaviours making them difficult to be detected. Consequently, termites have gained the reputation of notorious pests with an all-consuming appetite [2]. The reality is quite different: just 97 of more than 3,100 known species are considered to be economically relevant [3], [4], [5], with most species providing important ecosystem functions and are considered to be ecosystem engineers [6], [7], [8]. Due to their sociality, their inter-dependency, their ability to communicate and their strict organisation, termite colonies are referred to as superorganisms [9], [10], [11], [12]. Apart from having highly specialised direct communication based on vibrational information and pheromones [13], [14], termites build a variety of complex structures (underground nests, soil protruding mounds and nests high up on trees, cf. Fig. 1) as a product of cooperation [15] – presumably following simple sets of rules to produce a large diversity of shapes [16] through parameter tuning [17].

In 1954 Grassé [19] conceived that coordination during construction and excavation is achieved using stimulating patterns of matter for different regulatory responses including either (1) stigmergic stimuli, (2) responses to the environment or (3) nestmate interaction, factors which have largely been confirmed in research since then [20], [21], [22]. Small structures are designed based on the insect’s body size [23] while larger structures are built through collective interactions [15]. The environment, the state of the colony and the shape of the nest as well as the individual termite (caste, age, experience) determine individual and collective behaviours [24]. However, very little is known how these factors interact to affect mound size and variability, functional properties of different parts of the mound and among species and other probably important details, such as tunnel diameter and chamber size.^1^

The complexity, utility and potential sustainability of biological morphogenesis [25], especially nest construction, has inspired concepts of eco-friendly architectural designs [26], [27], [28], [29] and ideas of generating sustainable biocemented materials [30], [31]. Termite-built structures demonstrate how to protect the colony within a ’breathing’ shelter [32]; how fluctuations of intensive environmental parameters could be used to passively climatise architecture (*homeostasis*) [32], [33] of highest strength [19], [34] to generate all-year-round ideal living conditions [15], [35].

Noirot and Darlington [36] review termite nest architecture, climate regulation and defence, while Korb [37] studies similarly termite mound architecture, its function and construction with a focus on *functional shapes* of selected (mostly African) species. Consequently, past research was mostly concerned with autonomous nest constructions or building activities, the network structure of tunnels, and aspects of stigmergy and self-organisation [15], [28], [38], [39], [40], [41], [42], [43].

*Stigmergy*, hereby defined as *indirect* communication [44] to exchange information through *modification of the environment* [40], is a prerequisite of self-organisation and spontaneous order generation through local interactions of a seemingly erratic system [15], [45]. Swarm behaviour or self-organisation is part of autonomous systems research with its emerging domain of swarm robotics and artificial intelligence [40], [42], [44], [46]. In contrast, *direct* communication is provided by optical, pheromone, tactile and vibrational information [47], [48], [49]. Especially vibrational information (biotremology) has been a largely neglected communication modality, however, it is becoming increasingly clear that using vibrations is the dominant mode of communication in termites [13], [14], [50].

We hypothesise that biotremology is not only used to determine food size [50], [52] or to drum alarm [53], [54], [55], [56], [57] but could also be essential for the construction of termite nests. However, as indicated by Darlington [58], an explicit classification of various functional structural relationships between mound (nest, corridors and walls, material composition) and colony (individual, collective) is a neglected aspect in termite behaviour and ecology research. In addition to a brief review of well-known functionalities of termite nests, this paper is also aimed at discussing direct vibrational communications, as opposed to stigmergy, and identifying potential research areas which might offer insights into how termites interact within their mounds.

## 2 Classification of termite mounds and morphology

**Fig. 2:**
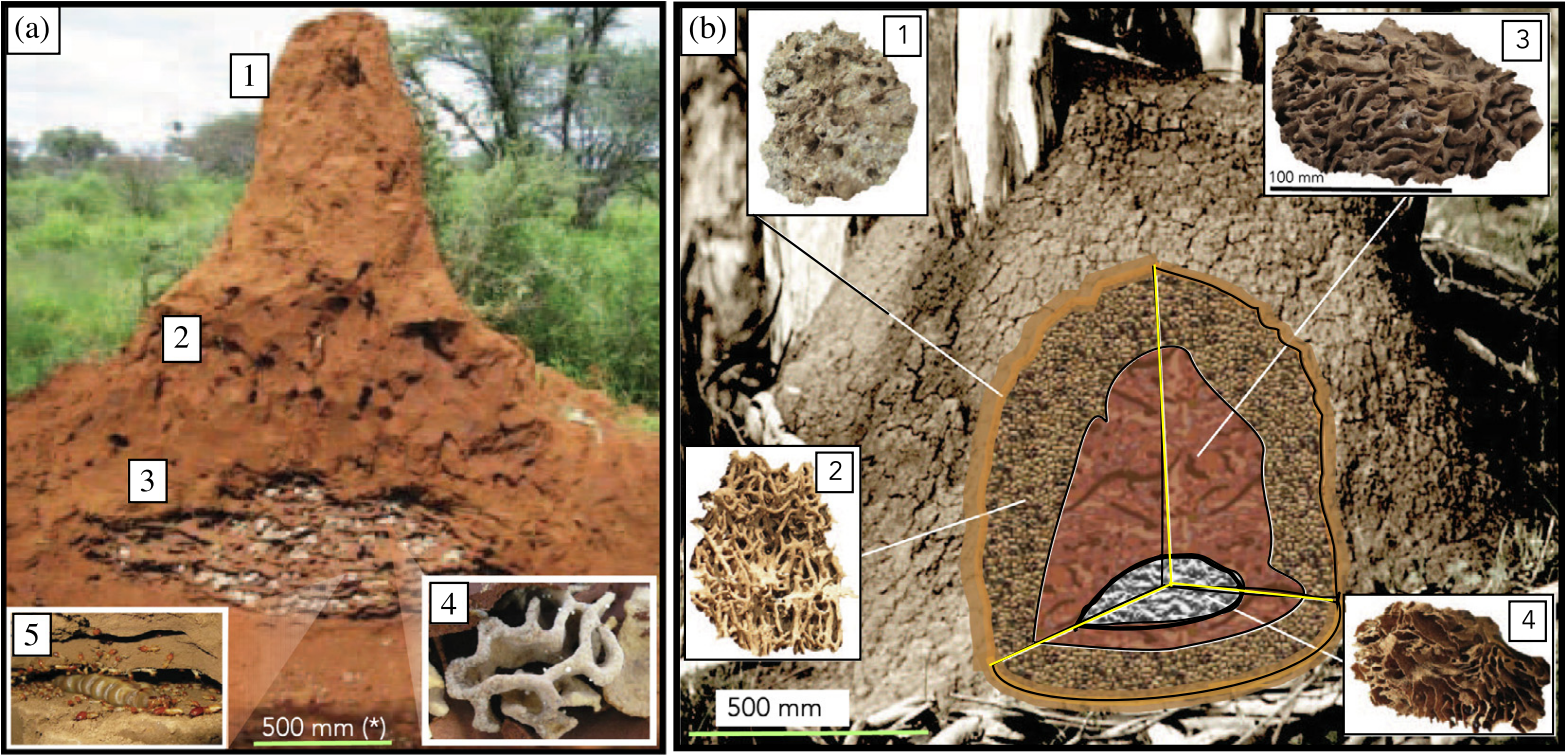
Termite nests of (a) *Macrotermes michaelensi* – 1 turret with apical hole, 2 conduits, 3 fungus combs with brood chambers, 4 fungus comb and nodules, and 5 queen chamber with queen, soldiers, minor and major soldiers (adapted from Grohmann (2010) [51], *estimated dimension); (b) nest of *Coptotermes lacteus* – 1 outer shell, 2 peripheral boxwork *periecie*, 3 nesting material, 4 carton material *endoecie* (Photo credits: Sebastian Oberst & Richard Martin, 2019; Tidbinbilla Nature Reserve, Australian Capital Territory, Australia).

Termite nests are made of an homogeneous *thermal envelope* – a hard outer shell as general protection, for defence against predators and protection against desiccation – and a heterogeneous *thermal inertia* [59]. 2 Fig. 2 depicts schematics of mounds of (a) the African termite *Macrotermes michaelensi*, and (b) *Coptotermes lacteus*. *M. michaelensi* is a member of the subfamily Macrotermitinae (the fungus-growing termites) in the family Termitidae or the *higher termites*. Many species of higher termites build mounds, of varying size and design; and much of the research into termite mounds has been conduced on *M. michaelensi*. It is African and is found primarily in savannahs, with complex nest constructions, including a turret which contains an intricate system of conduits for climatisation, fungus combs to decompose lignin and cellulose, nurseries and a central royal chamber [36], [60]. *Coptotermes lacteus* is a member of the subfamily Heterotermitinae, in the paraphyletic family Rhinotermitidae, one of the *lower termites*. It is of interest because it (and two other related Australian Coptotermes species) are the only lower termites to build mounds [61], [62]. It builds nests with a thick outer shell, followed by a complex peripheral boxwork, the nesting side and the brood chamber made of carton material [63]. While there are certainly common features, a classification of species according to those features has not been achieved to date, because of the following reasons.

### 2.1 About the structure of termite mounds

A systematic study of general principles of construction as found in termites of the savannah or the forest has never been attempted and it remains debatable whether general patterns and structures exist. Termite nest geometries and builds appear to be largely variable, even among the same species, but those in forests of the same species are on average smaller and more variable in shape and location than in savannahs [22], [60], [64]. The best explored structures, especially with regards to their ventilation system, are those of African species of the tropical savannah, namely the epigean nests of *Macrotermes* spp. and *Odontotermes* sp. (both Macrotermitidae), and in forests, specially *Cephalotermes rectangularis* (Termititdae, Termitinae). These mounds generally have an (undifferentiated) alveolar structure, with external shell and laminar internal structure [33], [36], [65]. The architecture of the outer shell is often finned like a radiator to facilitate large thermal gradients between the insulated chimney (apical hole [36]) and the outer shell and thin flutes [33].

The fungus growing Macrotermitinae are distributed from central Africa (e.g. *Macrotermes natalensis*) to southern Asia (*Odototermes obesus*); many species are mound builders, and many of these have been studied in some detail (see Fig. 2 (a)). These mounds may have either an open or closed, or variable outer shell and contain uninhabited conduits (multiple times larger than the size of a termite) to ventilate the nest [33]. The thermal inertia is composed of a peripheral boxwork *periecie*, which acts as an insulation layer. Inside of this is the central spherical nesting and the carton material *endoecie* for brood (hatchlings), which is for some species also used to store food^3^ The endoecie also contains the royal cells which house the queen(s) and king(s) of the termite colony [3], [36], [37], [65]. The periecie can open at the apex into a central air shaft as in the case of *Macrotermes natalensis* or *Macrotermes michaelensi*; the channel system is called *exoecie* if it is detached from the nesting structure as in case of *Tumulitermes* spp. [66]. Reaching out concentrically from the mound is a network of horizontal foraging galleries which sometimes appear prominently in photos as larger tunnel complexes, these appear to be different to the vertical ventilation conduits [36], [37]. However, the distribution of constructing macroscopic ventilation systems has never been studied to date. It seems plausible that parallels have developed and that the design of the climatisation systems of different termite species of different geographical origin would be of high interest from the evolutionary point of view, cf. [16]. Mainly due to the complexity of the task and of the structure itself, however, studying a diversity of species with local differences (geography, soils, temperature, different fauna), a systematic classification of termite mounds and their morphology has never been attempted. It should be noted that this complexity of mound structure may not be ubiquitous in all fungus growing termite species, or even in all *Macrotermes* species. In Thailand, *M. gilvus* mounds have a simpler structure, without obvious ventilation passages; indeed large colonies appear to occupy a smaller proportion of their mounds perhaps due to ventilation issues [67].

### 2.2 Nest categorisation and morphology

The oldest definitive fungus growing termite nests are from the Paleogen (ca. 31 Ma [68]), although complex structures interpreted as fossilised termite nests in the Clarens Formation in the Tuli Basin, South Africa date back to the Early Jurassic (181  Ma) [69]. Despite these great ages, fewer details are known about the evolution of construction principles of termite mounds as opposed to the evolution of termite species.

Different mound morphologies have emerged as a response to optimise the micro-climate, especially for rearing the brood, and to exclude predators (passive defence) [2], [36], [70], and can be found throughout trophic categorisation, i.e. for soil-feeders, soil/wood interface-feeders, wood-feeders, litter-foragers, or specialised- and incidental-feeders [71]. The ability of termites to modify their nest structure by tunnelling through it, e.g. by utilising clays, silts and sands, evolves as relative competitive advantage in co-existing species [72]. Consequently, morphologies of contemporary termite nests (termitaria) are diverse yet commonly categorised into being either hypogeal (subterranean, below ground), epigeal (above ground, protruding above soil) or arboreal (“tree-nest”), which can be within a cavity of a trunk or branch (become “pole-nests” for anthropogenic structures) or external to a trunk or branch.

Termites are hypothesised to have first used ’food as shelter’, as found for most of the basal termite families [36], [37], [65]; thus, the nest within the food (wood) significantly shaped the general colony parental care [73]. As first evolutionary step digging a network of subterranean galleries from this initial nest may have been allowed the colonisation of additional food resources. A second evolutionary step may have been the construction of distinct nests, as opposed to foraging sites, and a true worker caste [36]. Mizumoto and Bourguignon [16] suggested that a simple set of behavioural rules led to the exhibition of collective construction behaviour, especially to build shelter tube formations – an ability not observed in the sister group to termites, the wood-eating cockroaches in the genus *Cryptocercus*. Some species, such as *Reticulitermes flavipes* or *Reticulitermes grassei*, start as single-piece termite, then move on to other pieces thereby repeating evolution – the latter being also able to build internal walls [72]. Perhaps in a similar fashion, the evolution from tree-dwelling to mound-building can be observed in *Coptotermes acinaciformis*, for which the Southern form houses within the stem of a nesting tree, while the Northern form designs mounds adjacent to a foraging tree (Fig. 1 (a)) [61], [62].

The form of the mound of any species depends in part on the microclimate of the local habitat. As discussed by Korb and Linsenmair [60] (cf. [59], [74]), the appearance of the mounds of *Macrotermes bellicosus* differ from forests, where mounds have a smooth and thick outer wall, to those in the open savannah, where mounds have rough ridges and thin outer walls. Differences appear to be driven by humidity, shown experimentally by Carey et al. [75]. The porosity of the outer shell facilitates diffuse gas transport along concentration gradients while the small pore size makes the mound very resistant to pressure-driven bulk flow across its thickness with the mound surface acting like a breathable windbreaker [33]. The orientation of the mound relative to the sun is important as well [36]; e.g. Australian *Amitermes* spp. and *Tumulitermes* spp. build slap-shaped, North–South-oriented nests, for morning and evening sun energy intake for optimised heating in the cool of the twilight hours; however, the exact angle of orientation depends on the local conditions [37]. A similar pattern has been observed in *O. obesus* [33] and *M. michaelseni* [59] also.

Very little has been reported on the growth of termite nests, as small mounds made by young colonies are rarely encountered [76], [77], [78], [79], [80], [81], likely due to low survival of young colonies [82]. Instead almost all epigeal mounds in the field are mature (i.e. the colonies in the mound produce alates [83]) and usually skewed to the top end of the size distribution (mounds have been dated to be 100  years old, even 700  years old, with some estimates of thousands of years [84], [85], [86], [87]). Of course termite mounds most likely do grow with the termite colony population from nothing to the final, large size [88], [89], [90] – and likely in discrete stages [36]. The majority of the research on mound growth, however, has used only a modelling approach [22], [91], [92], [93].

For the few species with field data on mound growth, such as *Cornitermes cumulans* in South America and *Nasutitermes exitiosus* in Australia, the young colony occupies a small hypogeal nest which grows with the colony, eventually protruding above ground as an epigeal mound [22], [94], [95]. For the intermediate stage, nest complexity is assumed to be identical to that of mature nests [36]. In lower (paraphyletic) and higher (monophyletic) termites, nests are enlarged as required when the population increases; alternatively satellite nests are formed (polycalism) which offer enhanced dispersal through foundation by budding via neotenics [2], [32], [96]. The discrete growth of termite nests is thereby presumably a result of an immense inter- and intra-specific competition, as also evidenced in the fixed distance of locations of termite nests [64].

Humus (or soil) feeders found mostly in tropical forests or savannahs (e.g. some *Cubitermes* spp., *Anoplotermes* spp., *Apicotermes*-group spp., *Pericapritermes*-group spp., *Subulitermes*-group spp., all in the Termitidae), comprising more than a quarter of all termite species, often build hypogeal nests [3], [36], [97], [98], [99]. These nests have much simpler structures [36], a diffuse network of scarce galleries and cells (chambers) which form nodes (clusters of cells) within the soil, often filled up with larvae and nymphs [99]. The gallery system, merely plastered with faecal matter, preserves moisture and temperature [36]. Similarly, some soil feeders such as potentially *Nasutitermes eucalypti* or more often *Macrognathotermes sunteri* (both Termitidae), which are inquiline to and eat the mound of *Coptotermes acinaciformis*, tunnel corridors in nest portions of their host, which sometimes form discretely attached mounds, cf. Fig. 1(a) [100].

Some subterranean nests are limited by a continuous wall without openings but with a surrounding empty space, the paraecie [69], [95], which can be filled with pure and fine sand, cf. *Sphaerotermes* [101], or *Apicotermes* and Nasitutermitinae [36], [65]. The paraecie presumably enhances defence against predators, e.g. to exacerbate digging activities of ants [102], [103]. Epigeal nests start with a subterranean stage, before protruding to the surface. Epigeal nests may facilitate gas exchange and defence against predators in the soil but they are also more exposed to heat and predators above the ground (visibility). Arboreal nests often start subterranean or grow initially in a cavity of a tree before the colony moves (up the tree) to a newly built arboreal structure (e.g. *Microcerotermes biroi*, *Nasutitermes walkeri*, cf. Fig. 1(c)). As compared to hypogeal and epigeal nests, arboreal nest constructions require more energy, e.g. to transport building materials, but facilitate defence against larger predators (e.g. Tachyglossidae, Manidae [104], [105]). In case of severe damage of the main nest, arboreal species tend to migrate to specifically built surrogate nests [36].

## 3 Termite structures as functional materials

Termite mounds serve multiple functions: (1) Nest, nursery and food storage, (2) Thermoregulation and climatisation, (3) Defence, shelter and refuge, (4) Termite clay as building material and foraging tool, and (5) Stigmergy and communication channel.

### 3.1 Nest, nursery and food storage

The common digestion of plant fibres in lower termites is based on symbiotic interrelationships with various gut microorganisms (bacteria, flagellate protists, prokaryotic and eukaryotic symbionts [3], [106], [107], [108]) to digest lignocellulose, the major nutrient source of (the non-soil feeding) termites. Lignocellulose, composed of cellulose, hemicellulose (polysaccharides [109], [110]) and lignin becomes fermented by the protozoa (flagellates within the termites’ hindgut) under anaerobic conditions – with lignin being utilised in a smaller portion as energy source after fungus degradation [3], [111], [112]. Higher termites (Termitidae) usually do not require flagellates’ fermentation [110] and have a symbiont-independent cellulose digestion [3], [113]. Storing food within the inner nest’s carton material or other nest wall sections in the form of pre-digested lignocellulose (e.g. hemicellulose, xylose and galactose as in *Coptotermes acinaciformis* [109]) or cut materials [114], [115], [116] is widespread among termites, including many mound builders and grass-feeding species [3], [70].

The pre-digested lignocellulose may especially attract commensal species, inquilines or kleptoparasitic termites (mound-/soil- or humus-feeding species) such as *Macrognathotermes sunteri* found on *Coptotermes acinaciformis* mounds or *Cavitermes tuberosus*, on *Labiotermes labralis*, *Termes fatalis* or *Neocapritermes taracua* mounds, or *Inquilinitermes microcerus* found in *Constrictotermes cyphergaster* mounds (note a wide variety of termite species may inhabit opportunistically the mounds built by other termite species) [3], [117], [118], [119], [120], [121]. Some inquiline species feed on the already digested (pseudo-) faeces within the mound matrix of their host, e.g. mound feeders of the *Cubitermes*- (*Ophiotermes*-) group [3]; yet little is known about the exact source of energy for soil feeders and more research needs to be conducted, especially on species other than *Cubitermes* [108], [122].

Higher termites of Macrotermitidae make use exosymbiotic relationships to Termitomyces strains to break down lignocellulose, including lignin, by cultivating fungus in fungal gardens – an ability of the fungus due to co-evolution which made switching between multiple termite hosts difficult [106]. Nursery and fungus comb structure (e.g. insert in Fig. 2(a)) are built of finer particles (clay, fine silt), are carbon and nitrogen enriched as compared to foraging galleries [123] and are plastered with faecal layers to fight pathogens, missing in foraging or shelter tubes providing optimal conditions for the growth of basidiomycete fungi which grow to decompose plant fibres into nutritious compost which serves as food for the termite colony [32], [106], [124].

Other higher termites belong to non-fungus-farming species which grow complex structured bacterial combs by accumulation of pellets, as an evolutionary adaption of carton material to external rumen and which is assumed to allow the need for the removal of gut protist symbionts [125], [126]. *Sphaerotermes sphaerothorax* is a bacterial farming species which builds to two kinds of combs: the first one is made of an accumulation of faeces in the lower part of the subterranean nest (dark colour), the second kind is either made of mylospheres or buccal pellets; after bacterial action (e.g. spiral bacteria), *Sp. sphaerothorax* consumes the fermented (lighter coloured) pellets [125], [126].

### 3.2 Thermoregulation through ventilation

Termites are known for their thermoregulation, but only a few examples have been systematically studied (e.g. Macrotermes, Odontotermes, Cornitermes). Especially for those species homeostasis, i.e. air-exchange, temperature and humidity control via ventilation is important for the development and against desiccation of the colony, especially that of the brood (immature instar stage) [3]. Homeostasis relies on diurnal temperature oscillations, specific geometry (central duct and peripheral conduits), heterogeneous thermal mass (thin conduits, thick walled inner chimneys) as well as macro- and micro-porosity [33], [60]. In *Macrotermes bellicosus* the inner nest – the coolest part during the day [33] – is kept at a constant 30 ^°^C with a humidity “near saturation” [60]. The air exchange also impedes the spread of epizootics alongside antibacterial and spore germination inhibiting faecal pellets [70], [127], [128], [129], [130].

The epigeal nests of African and Asian *Macrotermes* spp. and *Odontotermes* spp. are considered either open or closed ventilation systems [35], [131], [132]. The mound shape, as well as internal geometry and whether ventilation systems are open or closed, depends on the night-day cycles, the sun’s intensity, the geography and many yet largely unknown factors; e.g. for *Macrotermes bellicosus* the ventilation system is *closed* in West Africa Guinean region, but *open* near the base of the mound in Uganda, Congo and Western Kenya [36], [60].

Open systems exchange gas through air flow velocity differences, either caused by steady forcing (e.g. convection currents due to metabolism) or by transient processes (diurnal driving, wind) [33]. Closed systems rely on diffusion processes between the interior and the exterior wall [60]. Primitive species (e.g. one-piece termites), exchange gas and humidity only via diffusion through the pores of the wooden nest [3], [36] while nests of the African higher termite *Trinervitermes geminatu* have closed, micropore-perforated outer walls for efficient gas exchange and water drainage [35]. Due to these advantages, it is possible that other species have air movement and climatisation in their mound (but there are likely species without, see [67]).

Thermoregulation in a termite mound is interesting due to its working principle. Similarly to a thermosiphon (evaporative cooling) [36], the gas exchange relies on the bulk flow within the nest and not on diffusion processes [33]. Thermal siphoning is internally driven, and happens mostly in the night [60]. Termites collect water in their water sacs (attached to the salivary and labial glands) [70], [133], [134], [135] for deposition onto their porous nest walls. As a consequence, warm air rises up the central shaft and descends in peripheral conduits [3], [36], [37], [136]. Temperatures of the peripheral air conduits within ridges are lower than those found in the central shaft; descending air exchanges respiratory gases through the ridges similarly to lungs, cf. [33], [59]. Peripheral air conduits in near-vertical orientation, often circular or broad oval in cross-section, have smooth surfaces to reduce air vortices forming and are large in proportion to a termite's body length [36]. Externally driven ventilation, by ambient temperature and local heating of the mound surface, is active during the day. The sun heats the peripheral mound, which causes convection currents; the air flow in the ridges leads to an increased CO_2_ diffusion and air rises upwards in the channels directly behind the outer wall and downwards through the central shaft [60].

The metabolism of termites in the mound plays an important role in gas circulation and thus for the thermoregulation in the mound. The production of metabolic gases has been well studied with estimates of gases corresponding with colony size of intact mounds in the field, cf. [35], [36], [60], [137], [138], [139]. While termites, as major biomass decomposers, are estimated to be responsible for up to 3% of the worldwide methane production [140], caused by *methanogenesis* in their hind gut, up to half of the emitted ${\mathrm{CH}}_{4}$ is reduced by microbial ${\mathrm{CO}}_{2}$ oxidation – catalysed by methantrophic bacteria living within the nest walls making the mound a functional “biofilter” [139]. The well-connectedness of the corridors facilitates a change in direction of the air circulation.

Termite mounds may experience high concentrations of ${\mathrm{CO}}_{2}$, which fluctuates daily and seasonally, due to the microporosity of the external shell. The mounds of *Odontotermes obesus* in Africa have ${\mathrm{CO}}_{2}$ concentrations of up to 6% during the day, those of *Macrotermes michaelensi* and *M. subhyalinus* average ${\mathrm{CO}}_{2}$ concentrations of about 3%, with reduced concentrations during the night from higher convective flows (−2.8 cms^−1^) [33]. These very high ${\mathrm{CO}}_{2}$ concentrations are due to termites and fungi metabolising in the same space. Termites without fungi may have lower concentrations, such as *Coptotermes lacteus* with around eight times normal atmospheric ${\mathrm{CO}}_{2}$ [141].

Termites exhibit high tolerance to fluctuations of gas composition and to very high concentrations of carbon dioxide (and low oxygen levels) which would easily narcotise other insect species (up to 20% before anaesthesia occurred as in the dampwood termite *Zootermopsis nevadensis*) [142]. While methane production is stronger in fungus growing termites, the gut protozoa and associated fermentation processes produce large amounts of metabolic gases in most other termites as well and it may be assumed that similar high tolerance is widespread in this taxa.

### 3.3 Defence, shelter and refuge

Defence is often assumed to be active, as a reaction of a defender (termite soldiers or workers) against an opponent, such as intruding predatory ants [143] by making use of mandibles, chemicals, sticky or toxic secretions or even suicidal bombing (autothysis) as found in *Neocapritermes taracua* [36], [47], [144], [145], [146]. The stickiness of secretions has an immediate effect on attackers as compared to slowly acting lethal toxicants [147] and might also affect more than one individual. However, in some higher termite species, such as *Apicotermitinae*, the termite soldier caste is even lost [36] since for termites, direct confrontation is only a last resort.

In fact, most termite defensive action originates from passive means by staying camouflaged (including avoidance of detection [crypsis] and recognition [masquerade] [148]) and cryptic, hidden and being protected by the mound and clay galleries or blocking passageways using e.g. sclerified heads [34], [36], [70], [149], [150]. Other geometric constraints are narrowing corridor systems to allow only termites to pass through one at a time such as those often found close to the royal cells [36] or also material partitions advantageous for the royal pair [151] for the drywood termite *Incisitermes minor*. *Prohamitermes mirabilis* uses prefabricated plugs made from a small foreign particle (sand grain) wrapped with nest cement, but larger than the entrance to the chamber, to seal off nest chambers which commonly only have two small holes in the side walls [147].

Camouflage is the most prevalent *passive defence mechanism* in termites and encompasses general concealment or disguise, including morphology and materials found in the environment, acoustics and vibrations, smell and vision, hindering detection or recognition [148]. Here it is important to consider the predator–prey, host-inquiline, relationships and the concealment of information related to senses – more specifically the perceptual mechanisms involved (*natural camouflage*), which is far from intuitively obvious [148]. Visual detection of termites in the nest becomes infeasible as they live in the dark, so that other signalling modalities have to be exploited, such as tactile information, acoustics and vibration [70]. As shown by Oberst et al. [13], termites conceal their activity by causing a minimum of noise, thereby avoiding ants – their main predators [147]. On the other hand, termite nests such as those of arboreal *Nasutitermes* spp. are perfectly adapted in coloration to the underlying stem, resembling in shape bizarre lumps as found after a trauma of the tree as a consequence of a healing process, in burls (triggered genetic predisposition) or as a consequence of certain (fungal) tree diseases (e.g. black knot) [152].

However, the tunnel system structure and the tunnels' lengths are influential in the effectiveness of defence of a colony. Outside the nest, termites invest lots of energy to stay cryptic within their gallery system and use soil bioturbation against predation [8]. By measuring the lengths of all possible paths within a *Cubitermes* sp. nest using methods from graph theory, paths were found to be much shorter than would be expected if adjacent chambers were simply randomly interconnected [38], [97]. The connectivity of a computer model resembled that of the scanned termite mounds, which represents a compromise between efficient connectivity (large network) and ease of defence against intruders (fewer connections) [38].

### 3.4 Termite clay as building material or foraging tool

Termites use different building materials in their mound and galleries with a range of properties [34], [70], [123]. As noted by Grassé, termites (*Sphaerotermes sphaerotorax*) use homogeneous mixtures of clay and fine sand particles, which is a different composition from the coarser soil around the nest [19]. These observations have been replicated many times, so that by building galleries for foraging below ground and translocating large soil quantities on the ground for harvesting litter, termites function as bioturbators on the profile level and soil aggregate reorganisers at the scale of soil microaggregates (from 50 to 250 μm) [8], [153], [154]. Epigeal or arboreal nests of *Labiotermes labralis* are alveolar carton material nests, strong, robust and heavy, and all nest parts can contain visible traces of sand, while hypogeal nests of *L. longilabius* are composed of tiered, flat chambers, a recurrent morphology in Syntermesinae nests [65], [155], [156].

Arboreal nests consist of exogenous lignocellulosic materials (plant matter, including being pre-digested via enzymatic secretions of salivary and labial glands, and faeces) and have fewer inorganic components [96], [157], [158]. Hypogeal nests are more similar to epigeal mounds and made of clay and lignocellulosic materials. Soil components such as granulated clay and clay silicates, (fine) silts and sands are used for the outer wall of epigeal nests with smaller proportions for the nursery [33], [34], [36], [70], [123], [158]. The moulding of extra organic matter (carbon, nitrogen) into a clay/silt matrix may be assumed to increase the mechanical strength of termite structures [30]; but more research in this direction is required. Also, owing to the faecal and saliva content, termite pellets (boluses [30]) are antibacterial and anti-fungal as studied extensively by Chouvenc et al. [129], [130].

Especially for the royal cell, clay soils from deeper layers are the preferred building material due to their greater water-holding capacity [36], [123]. Shrinkage decreases in clay-enriched termite soils (*Macrotermes bellicosus*) and its aggregate stability after heavy rainfalls increases [158]. *Odontotermes nr. pauperans* (Macrotermitidae) prefer fine silts and clays for fungus comb walls and inner nest gallery construction over coarse silts and sands as found in top soils [8], [30], [158]. Mound walls in Northern Australia for the mud-nesting ant *Polyrhachis sokolova*, Forel (Hymenoptera, Formicidae) are often composed of soils from non-surface soil layers including 47% kaolinite (at ca. 228  mm depth), 23% boehmite (at ca. 315  mm) and 10% gibbsite (at ca. 110  mm) [159]. Eggleton and Taylor found that for the soil composition on the Weipa Bauxite (northern Australia) frequent wildfires dehydrate gibbsite to boehmite or alumina in bauxite fines (particle diameter <75 μm) which is used by termites for above nest construction [160]; presumably this necessitates constant renewal of the clay in the mounds, up to depths of 20 m. Other than that, there is no review detailing the soils from which termite mounds are composed; in fact, the mounds of just a few species have been investigated (see references above).

Termites have been found to dig much deeper. There has been some interest in using epigeal termite mounds for sampling minerals found at greater depths, especially for gold exploration. Sampling termite mounds on the surface is considerably faster and cheaper than drilling, cf. [161]. Termites have been found to tunnel to depths as great as 70 m, although this is highly variable [162]. *Tumulitermes tumuli* mounds contain gold particles found at 1 m to 4 m depth [163], [164]. Deep soil components (clay, fine silts) are better suited to the nest chamber construction for water retention (free water and adsorbed cationic water [30], [165]) and require reduced carbon and nitrogen supplements compared to topsoils [123] which are the preferred, less laborious alternative for galleries. Some termite species such as *Nasutitermes longipennis* build the external part of the nest in “sand and clay” cemented with *stercoral mortar* [166], while the nest chamber is built of paper-like material. This carton nest is composed of a mixture of faecal matter and wood fragments, darker in its appearance and composed of a larger amount of organic matter [109], [167], resembling cardboard or paper-mache [168].

Kandasami et al. [30] studied the mechanobiological effects of cementation of bioadhesives in structures of the fungus-growing termite *Odontotermes obesus*. Boluses (soil-pellets [40]) are up to 1.2 mm ball-shaped “termite bricks” formed of soil particles accumulated by individual termites [30]. Boluses include glandular secretions (saliva with cellulose digesting enzymes [109], [169]) and difficult to digest lignin-based phenolitic excretions [170], [171]), suggesting a wide range of cementation abilities symptomatic for an increased organic content of the mound soil [109], [172]. The epigeal density of the soil was estimated to be 1.42 to 1.68 g/cm^3^; in the horizontal direction, no difference in soils used could be found and the material can be classified as homogeneous [30]. The mean particle size of the mound was about 6 μm (with a mean moisture content of 17%) compared to a 20 μm mean particle size of the surrounding soil.

Overall, the porosity of the outer wall of termite mounds decreases but the microporosity of less than 0.1 μm pore size (5 μm [33]) increases, reducing the ability of water to penetrate the soil efficiently [158]. The resistance to bulk flow pressure [33], due to small pore size, decreases susceptibility to erosion and collapse [30]. Macro-porosity varies from 37% to 47% for *Odontotermes obesus* [30], [33]. Using a CT image of a termite mound cross-section the macro- and microporosities of *Microcerotermes nervosus*, *Macrognathotermes sunteri* and *Tumulitermes pastinator* were estimated to be roughly (24%, 19%), (36%, 35%) and (39%, 23%) respectively [138]. Porosity in different scales is related to fractal structures found in nature [45]. The fractal dimension of a surface model of the mound’s shell was estimated to be 1.88, 1.91 and 1.93 using the Bouligand-Minkowski method, indicating multiple scales and fractality [138].

Termite constructures of clay and lignocellulostic cement can have considerable strength, in mounds but of particular interest are other applications. For example, *Coptotermes acinaciformis* builds structures in a dynamic process as a foraging tool to reinforce load-bearing clay walls to access otherwise inaccessible food [34]. The compressive strength for termite-built load supporting structures in *Coptotermes acinaciformis* to hold a specific load was about 0.22 MPa [34]. Impressive as this is, *Odontotermes obesus* mound walls are up to 1.8 MPa for the outer shell [30], perhaps to deter vertebrate predators, such as aardvark and pangolins [173], [174], [175]. Termite mound materials have been considered for use in termite construction [176], [177], [178], [179]. This amazing behaviour of termites to build load-bearing structures is complemented by their ability to manipulate moisture in wood to avoid buckling [70]. However, relatively little is yet known about the exact mechanisms involved.

### 3.5 Stigmergy and Biotremology

In his seminal paper, Grassé (1959) [19] described stigmergy for the first time as a paradoxical phenomenon of individual insects behaving in a decentralised way, but building structures as if being centrally organised; stigmergy is a class of mechanisms that mediate animal-to-animal interactions [46]. Since thousands or even millions of individuals build complex mounds, self-organisation is assumed to play a decisive role in termite colony organisation [15], [23], [180], [181]. Positive and negative feedback processes [46] (e.g. internal airflows, transport mechanism) lead to a decentralised optimal construction of a functioning mound [15], [33].

Stigmergy can be subdivided into qualitative (self-assembled dynamics) and quantitative (self-organised dynamics) aspects [181]. Traces left and modifications made by individuals in their environment may feedback on them (indirect communication) [46]. Local interactions of simple agents produce complex spatio-temporal structures using nonlinear amplification of heterogeneities and other fluctuations [40].

In many early studies on stigmergy based on ants, pheromones and optical cues [48] are considered to be the trigger for collective action [22], [23], [35], [180], [182]. Hence pheromones have largely been held responsible for providing the cues required for quantitative stigmergic constructions in termites, e.g. soil pillars or stripes are built, after an initial non-coordinated (random) individual action of insects, in a coordinated way using pheromone impregnated pellets [22], [23], [182]. Qualitative stigmergy is the response to a stimulus independent of its concentration and which allows switching between different behaviours as triggered by varying cues.

The processes of termites building have been studied by Deneubourg [183], Bonabeau et al. [182], Feltell et al. [40] and Khuong et al. [23], mostly using sets of partial differential equations to describe reaction–diffusion, stochasticity, dynamic self-organisation or adaptation. Diffusion processes originate from cement pheromones (also called *construction pheromones*), which are supposedly left in boluses and act as short range navigation feedback while trail following pheromones act as long range navigation feedback; random walk processes are caused by termites staggering off [40]. However, pheromones are likely not the only source of information for termites. Ocko et al. [22] suggested a mathematical model to test morphological diversity of termite mounds by coupling environmental influence with social behaviour: advection and diffusion of heat and pheromones through a porous medium are modified by the mound’s geometry and also influence the geometry through termite behaviour. Recently Calovi et al. [44] showed convincingly that topological cues can provide a long-term physical memory of building activity that pheromones alone cannot provide: in laboratory experiments soil displacement (initial termite positioning and building activity) was positively correlated with surface curvature but not with inclination or height. Green et al. [184] showed that excavation and worker aggregation, rather than a cement pheromone, are sufficient to trigger self-emerging termite constructions.

There is increasing evidence that termites us other information. Not visual information as termites are blind and there is no evidence that termites perceive drumming signals via airborne sound [149], [185], [186], [187]. Termites have been found to communicate complex information using micro-vibrations (biotremology), an archaic and largely neglected, signalling modality [13], [48], [50], [57], [188], [189]. A communication signal’s active space consists of the source; a transmission medium; and a receiver [150]. Modes of vibrational communication in termites consist of (head, postmomentum) drumming as vertical oscillatory movement (11 Hz to 16 Hz repetition rates [13], [14]); longitudinal oscillatory movements or tremulation (jerking, jittering); or using complex oscillatory movements which combines vertical and horizontal movement presumably with releasing an odour [14].

## 4 Discussions

As outlined above, there is variable depth of the state-of-the-art knowledge about the various functionalities of termite structures and there is a lack of systematic studies to allow general features and differences to be classified. Here we will discuss and identify key research topics that will potentially answer the question on the holistic picture of the interrelationship between termite structures, termites and their behaviour as a superorganism.

While bees or ants would survive without their nest for some time, termites would be exposed to the twin dangers of desiccation or predation [13]. Similarly, without a termite colony, the mound would become brittle, and collapse like a ’house of cards’ [34]. Termite nest architecture is therefore an expression of innate insect behaviour, altered by contact with the environment as “morphological expression of the sum of behavioural patterns” [3], [36], [151]. Thus, past and contemporary research largely expanded on how termites (mostly African Macrotermiditae) climatise their mound; how colonies organise chores, decentralisticly and autonomously, assuming stigmergic and self-organisational mechanisms as root cause of complexity and collective building [15], [42], [46].

*Pheromones and self-organisation revisited.* The building process rather than the built structure has been the centre of interest [46], [181]. Termite tunnelling has been mathematically modelled using (reaction-) diffusion systems, Laplacian growth models or Gaussian processes (diffusion system with randomised initial conditions), yet it is unclear to which scale these simulations are valid as no complete experimental validation is provided [15], [46], [190]. Corridor systems appear tree-like, as e.g. found in *Cubitermes* spp. [38], [97], containing only few loops. King et al. [33], however, described corridors and conduits as *well-connected*, the essential enabler to successfully use gypsum in endocasts. The connectivity of the tunnels and nodes (chambers) is attributed to a sub-function of defence or climatisation – the assumption that the tunnel system and digging activities follow diffusive processes or resulting in tree-like shapes seems to be a convenient simplification, the truth might yet lie somewhere in-between, with stronger emphasis on *determinism*, i.e. defined functionality of engineered structures.

While there is largely consensus that group level patterns emerge from interacting individuals following simple behavioral rules (individual-collective behaviour interaction), stigmergic building processes presumably originate from a cement pheromone. Yet, since termite mounds seem to grow in discrete stages, it has been argued, that a general continuous (global) growth model based on molecular diffusion of pheromones through the mound wall can be excluded [22], [64]. A pheromone is assumed to be embedded within termite boluses and taken as main factor for diffusion processes with randomness being induced e.g. through termites walking off the construction side [15], [16]. However, no cement pheromone has yet been identified [44], [191] so that Green et al. [184] suggested a chemical signal other than a pheromone. What if a largely unknown mechanism, different to stigmergy but related to pheromones, is responsible for building?

*Biotremological signals.* Recently, the action of digging and the aggregation of termite workers have shown a strong effect on recruiting termites for excavation and building work [184]. Aggregation alone *as information*, though, cannot be the only factor since termites within the nest walk and live next to each other which also leaves traces and signals [13]. It is also mentioned in [44] that termites act as physical obstacles and therefore limit the excavation. However, if termites are blind, cues other than aggregation and excavation could be the trigger for increased building activities. The application of *Random walk* or *swarm behaviour* models, widely applied in computer science, seem debatable in light of the eusociality of termites, which follow explicit cues and directed signals [13], [15], [46], [48], [192]. Some of the most prevalent direct signals termites are exposed to are those they use for biotremology, yet near to nothing is known about how termite colonies communicate in detail using vibrations such as using their mound as a communication channel, being adjacent to colonies of the same species or other species (intra- and interspecific communication among strongest inter-and intraspecific competition).

Grohmann et al. [64] assigned regular mound distribution patterns and colony size of *M. michaelseni* to intraspecific competition for foraging areas; it may be assumed that communication and eavesdropping are significant in colony survival. Evans et al. [193] studied how the subordinate drywood termite *Cryptotermes secundus* eavesdrops on the dominant subterranean termite species *Coptotermes acinaciformis* to choose smaller pieces of wood to avoid competition; similar strategies – a preference for distinct diets to avoid conflicts – have been found in many neotropical termite cohabiting builder- and inquiline-species-relationships, cf. [120]. Oberst et al. [13] found that termites of the commensal species *Macrognathotermes sunteri* are very quiet, and their walking cause less vibrations than its host species, *Coptotermes acinaciformis*, resulting in the so-called *disguise in the form of insignificance* as a special mechanism of camouflage [148], [194]. Similar relationships, whether they are host-commensurate/inquiline or parasite relationships, are known in many South-American species [195], however, whether signalling is based on mainly chemical or vibrational signals/cues or on multimodal effects, needs to be yet studied for each relationship separately.

Considering that biotremology plays a central role in termite communication [13], [14], [49], [50], and that the corridors within the mound are likely to be saturated with pheromones, and cannot work as a two-way communication system due to the directed airflow within the tunnels, the use of substrate borne vibrations and synchronisation seems advantageous. Synchronisation, as studied in nonlinear dynamics and mathematical physics [45], [196], [197], as deterministic oscillatory (here: vibratory) motion, is observed in both the physical and biological world, ranging from mechanical oscillators and bio-acoustics to predator–prey cycles and ecosystem dynamics [196], [197], [198], [199], [200].

Synchronisation of vibrational information might be more important to building and nest growth, triggered initially via localised individual action which may lead to global collective behaviour. The termite nest would act as a both a communication network and a large vibration sensor; locally constrained, vibrational signals and cues would provide subnetworks with synchronised tasks clearly defined via transmission through the structure. Studying the mechanical composition (type of clay, silt and sand particles used) and their compound properties would enable a deeper understanding of how termites modify their surroundings – which should be crucial for their eusocial character and the information exchange (from nestmate interaction to synchrony) required to run a colony of several million individuals.

Since biotremology has been shown to be significant in termite colony organisation, local and global synchronisation rather than stigmergy could be the prevalent trigger for building activities and the reason for group-level pattern emergence; this remains to be determined [14], [34], [70]. In order to study the mound and the structure of a termite nest, its wave propagation, its filter properties and its function as communication channel or even as a communication network, the material properties of the entire structure need to be determined.

*Understanding the structure*. Until recently the galleries of ant and termite nests have been studied using endocasts, e.g. gypsum, dental plaster or lead [32], [33]; however, novel technology using X-ray and specifically micro-computed tomography (μCT, mm range) now allows non-destructive visualisation of tunnels and details of the structure [38], [151], [201]. The ventilation of the mound as well as the emergence of tunnel systems and their mathematical descriptions has received much attention followed by study of the coordination of individuals and their collective behaviour using conventional statistical and Fourier-based methods [15], [35], [43], [202]. Yet, medical imaging lacks resolution and classical Fourier-based methods are linear and neither cover the spatial nor the temporal character of termite-built structures. Sophisticated measurement techniques such as ultra-high- or super-resolution X-ray tomography imaging, atomic force microscopy, alongside accurate granulometry, spectrometry, excellent computational resources, novel big data analysis techniques and computational intelligence methods would be required to capture the microscale of the walls including their porous structure and multiscale material characteristics and compositions. We know that variations in lignin characteristics and density fractions of termite nests reflect differences in feeding guilds of the studied taxa [170]. However, the exact composition of termite-built structures including the kind of lignin-based phenol used in different parts of the mound considering different functions remains unanswered.

Cation-adsorbing capacity provides “expandable clays” as a surface chemical or surface complexation process to facilitate the exchange of chemical species between an aqueous solution and mineral surfaces present in geological porous formations [30], [165] which could be related to micro-porosity, ventilation and natural evaporative cooling. Yet to-date, there is no clear understanding on which material composition can transmit signals efficiently, to carry loads, and to store which kind of food; there is virtually no knowledge on the geometry of the structures termites build (the tortuosity of the corridors or the porosity of the walls) and their effect on the ventilation; air-conduits are supposedly smoother than other parts of the nest [36] – however, different surfaces can cause the fluid’s boundary layer to change and the effect on ventilation should be quantified.

The techniques used to visualise the nest as well as analysis methods applied to study complex structures have been identified as being problematic [15]. Since data of natural phenomena are inherently complex, nonlinear time series analysis (NTSA), particularly recurrence plots and their quantification measures as increasingly applied in science and engineering, could provide valuable insights into the physics of termite-built structures [203], [204], [205], [206], [207]. While these methods have been foremostly applied to the understanding of complex time-dependent behaviour, they are in general also applicable to discontinuous-discrete or continuous spatial and temporal-spatial structures [45], [161], [207]. Using NTSA measures to determine whether the wall composition and the tunnel geometry avoid being detected by ants in coexistence with termites as observed in [13] could be an interesting area of research. Using machine learning tools would allow features to be extracted and spatially different structures to be classified for species analysis so that evolutionary and ecological traits in their structures may be identified. However, as indicated by Korb (2011) [37] there are still too many open questions about the material properties of the walls, the multiple functions of structures and their connection to individual behaviour and communication, that can only be answered by highly multi-disciplinary studies.

## CRediT authorship contribution statement

**Sebastian Oberst:** Conceptualization, Methodology, Investigation, Funding acquisition, Resources, Writing - original draft, Writing - review & editing, Visualization, Project administration. **Joseph C.S. Lai:** Conceptualization, Writing - review & editing. **Richard Martin:** Writing - review & editing, Visualization. **Benjamin J. Halkon:** Writing - review & editing, Funding acquisition, Resources. **Mohammad Saadatfar:** Writing - review & editing, Resources. **Theodore A. Evans:** Conceptualization, Writing - review & editing, Funding acquisition.

## Declaration of Competing Interest

The authors declare that they have no known competing financial interests or personal relationships that could have appeared to influence the work reported in this paper.

## Figures and Tables

**Table 1:** Extraction of species of the order Blattodea investigated within this mini-review and sorted according to their phylogeny from primitive (subsocial, wood eating cockroaches *Cryptocercus*) to basal, subsocial termites (Kalotermitididae) to more advanced, eusocial species [118], [208]; mound size (max size: Height [m]/ Diameter (ø) [m] / Depth [m]; ${ø}_{C}$ - average corridor diameter; (i) basal termite, (ii) lower termite, (iii) higher termite; N. = North, S. = South; * guesstimate, e.g. from photograph, insufficient information provided; TBD - to be determined.

Species	(Sub)family	Origin	Nest/food	Mound size	Comments
(1) *Cryptocercus*	Cryptocercidae	N. America	none/wood	within wood	subsocial, wood-eating cockroach; [209]
(2) *In. minor*	Kalotermitididae, (i)	Asia	wood/wood	1-piece log	tunneling;entrance 1.9mm [151]
(3) *Cr. secundus*	Kalotermitididae, (i)	Australia	wood/wood	1-piece log	only tunneling; [193]
(4) *Zo. nevadensis*	Archotermopsidae, (i)	N. America	wood/humus	1-piece log	no true worker caste; [210]
(5) *Re. flavipes*	Rhinotermitidae, (ii)	N. America	mound/wood	TBD	starts as 1-piece termite; no internal wall; [209]
(6) *Re. grassei*	Rhinotermitidae, (ii)	N. America	mound/wood	TBD	cf. *Re. flavlipes* + internal walls; [209]
(7) *Co. acinaciformis*	Rhinotermitidae, (ii)	Australia	mound,tree/wood	1/1.1/0.6	S.: *tree-nesting*; N.: *mound-building*; [34]
(8) *Co. lacteus*	Rhinotermitidae, (ii)	Australia	mound/wood	2/2/0.7	thick clay-like walls see Fig. 2; [63]
(9) *Na. exitiosus*	Nasutermitinae, (iii)	Australia	mound/wood	ca 0.6/0.6/0.3	soft shell/carton mat., hard interior; [211]
(10) *Na. walkeri*	Nasutermitinae, (iii)	Australia	arboreal/wood	ø 0.7 m	brittle outershell; light weight nest; [18]
(11) *Na. eucalypti*	Nasutermitinae, (iii)	Australia	wood,soil/grass	TBD	nest in litter/wood; [100]
(12) *Na. longipennis*	Nasutermitinae, (iii)	Australia	mound/ grass	* 0.75/0.2/0.2	hard outer shell; [100]
(13) *Tu. tumuli*	Termitinae, (iii)	Australia	mound/ grass	0.6/0.2/0.1	hard outer shell; [163]
(14) *Tu. pastinator*	Termitinae, (iii)	Australia	mound/grass	0.02 m^3^	hard outer shell; [212]
(15) *Mi. nervosus*	Termitinae, (iii)	Australia	mound/	0.01 m^3^	small nest, carton; mineral; [3], [212]
(16) *Mi. biroi*	Termitinae, (iii)	New Guinea	arboreal/wood	77 liters	see *Mi. nervosus* but carton material; [213]
(17) *Ca. tuberosus*	Termitidae, (iii)	S. America	arboreal/ soil	inquiline to (18)(20)	irregular; concave trunk sect.; [119], [214]
(18) *La. labralis*	Syntermitinae, (iii)	S. America	mound/soil	110 liters = 0.11 m^3^	alveolar, carton & at tree base; [65]
(19) *La. longilabius*	Syntermitinae, (iii)	S. America	subterr./soil	TBD	tiered, flat chambers; [65]
(20) *Te. fatalis*	Termitidae, (iii)	Africa	mound/humus	12 ft	clay, 3-4 sugar loaf 1ft high turrets; [53]
(21) *Neoca. taracua*	Termitinae, (iii)	S. America	mound/humus	TBD	diffuse nesting structure, soil; [3], [117], [119]
(22) *Mac. sunteri*	Termitinae	Australia	mound/soil	34 liters = 0.034 m^3^	cf. Fig. 1(a); nests within wall of (6); [3], [138]
(23) *A. meridionalis*	Termitinae, (iii)	Australia	mound/grass	4/2.5/1	cf. Fig. 1(b), ridges/sheets N-S direc.; [3]
(24) *Pr. mirabilis*	Rhinotermitidae, (iii)	Asia	subterr./ humus	ø 0.3 m	peat ground, cylindrical; [147]
(25) *Od. obesus*	Macrotermitinae, (iii)	Africa/Asia	mound/fungus	3/6/1	hard, outer shell, ridges, turrets; [30], [33], [40]
(26) *Ce. rectangularis*	Termitinea, (iii)	Africa	mound/humus	1/1/1	hard shell around *habitacle*; [33], [36], [65], [117]
(27) *Si. mushae*	Termitinae, (iii)	Asia	subterr./soil	TBD	diffuse galleries; [171]
(28) *Cu. fungifaber*	Termitinae, (iii)	Africa	arboreal/soil	cm:8.5/33/(3*);1792cm^3^	øC:<1 mm (*); [38], [97]
(29) *Sp.sphaerothorax*	Sphaerotermitinae, (iii)	Africa	subterr./wood	TBD	bacterial farming, polycalic; [125], [126]
(30) *Tr. geminatu*	Termitinae, (iii)	Africa	mound/grass	0.35/0.35/0.2	shell 11.1-14.9±4.9-5.6 mm; [35] inner wall 7.2-7.7±2.6-3.3 mm
(31) *Ma. michaelensi*	Macrotermitinae, (iii)	Africa	mound/fungi	3-9/4-5/1-1.5	cf. Fig. 2(a), hard outer shell; [32]
(32) *Ma. natalensis*	Macrotermitinae, (iii)	Africa	mound/fungi	*5/10/1.5	hard outer shell; [36], [37], [163]
(33) *Ma. subhyalinus*	Macrotermitinae, (iii)	Africa	mound/fungi	1.55/3/0.5	hard outer shell; [215]
(34) *Ma. bellicosus*	Macrotermitinae, (iii)	Africa	mound/fungi	1.83 to 5.41 m^3^	hard outer shell; [216]

## Appendix A Overview table

Table 1 provides an overview table of species taken as examples in this mini-review. The table is sorted according to the phylogeny adapted from [118], [208], from more primitive ”basal” species (starting with Cryptocercus, a wood-eating cockroach) to higher termites. While mound sizes are sometimes documented, there is generally no range nor average value given; the data as provided in the table for some is estimated from (old) photographs, without explicit scale. Wall thicknesses and other details of built termite structures, such as corridor width, have not been studied systematically but have been explained where applicable in the main body of the manuscript. Physical properties of the nest wall, functional significance of differently created structures and dimensions of various functional parts have not been systematically studied so far. Therefore, data provided in the table are built on very sparse information and largely more of qualitative nature, even if numbers are provided.

## Footnotes

1 An overview of termite species exemplified in this review as well as their mound sizes and other comments related to their nests can be found in Table 1.

2 The terms such as “thermal envelope” and “thermal inertia“ were developed for termite mounds in savannah and grasslands, which are exposed to high levels of insolation and wide daily and seasonal range of temperatures. These terms may be less relevant to termite mounds in closed canopy forests, which are exposed to low levels of insolation and a narrow range of temperatures.

3 In *M. natalensis* the nest shows radially inwards pointing fungus comb gardens (exosymbiosis with *Termitomyces*).
